# Use of Clinical Video Telehealth as a Tool for Optimizing Medications for Rural Older Veterans with Dementia

**DOI:** 10.3390/geriatrics3030044

**Published:** 2018-07-30

**Authors:** Woody Chang, Marcia Homer, Michelle I. Rossi

**Affiliations:** 1 Division of Geriatrics, University of Pittsburgh Medical Center, Pittsburgh, PA 15213, USA; woodyc@alumni.stanford.edu; 2 Geriatric Research Education and Clinical Center, Veterans Affairs Pittsburgh Healthcare System, Pittsburgh, PA 15240, USA; marcia.homer@va.gov

**Keywords:** telehealth, dementia, medications, rural, veterans, Beers list

## Abstract

Community-Based Outpatient Clinics (CBOCs) allow delivery of primary care to rural veterans who are far from a main Veterans Affairs (VA) campus. However, CBOCs often do not have physicians with geriatric training. We used a clinical video telehealth (CVT) dementia service (Teledementia clinic) based in the Pittsburgh VA Healthcare System to optimize dementia patients’ medications and potentially inappropriate medications (PIMs). We analyzed 199 CVT patient encounters from 1 January 2016 to 31 December 2016 and compared different medication changes per encounter between the initial CVT consults and the follow-up visits for all medications and PIMs as listed in the 2015 Beers Criteria, to see if there was a decrease of each kind of change, which is being used as a surrogate for optimization. We found that initial CVT consults, compared to follow-up visits, had greater medications added (0.731 vs. 0.434, *p* = 0.0092), total overall medications changes (1.769 vs. 1.130, *p* = 0.0078), and the stopping of 2015 Beers Criteria PIMs (0.208 vs. 0.072, *p* = 0.0255) per encounter. The fewer PIMs discontinued and fewer medication additions in follow-ups implies that our patients’ medications tend to stay optimized between visits. The teledementia service represents a novel way to provide geriatric assistance to CBOC VA primary care physicians for rural veterans with dementia.

## 1. Introduction

It is widely known that there is a disparity of care between urban and rural patients. In fact, rural patients typically have higher barriers to access general and specialist medical care, leading to poorer outcomes as compared to their urban counterparts [1,2].

This is especially important with the management of dementia. The WHO estimates that there are 50 million patients worldwide with dementia with 10 million new cases annually [3]. Indeed, the costs of care are staggering. Just in the U.S. alone, it was estimated to cost $818 billion in 2015, increasing to $1 trillion as of 2018 [4]. In addition, home-dwelling dementia patients are more likely to live in rural than urban areas [5]. Being in a rural area bring many difficulties to providing medical care. Not only is there a barrier to care due to behavioral or psychological symptoms of dementia and frailty, but also the physical distances and poorer infrastructure in rural areas increase the cost and effort to provide the same level of dementia care as urban patients.

We also know that there is a strong correlation between dementia and increased risk of polypharmacy [6,7], with polypharmacy leading to poorer outcomes overall [8,9,10]. As the rural population is predicted to increase faster than the urban population [11], it becomes even more important for these patients to have access to geriatric specialist care that may better be able to handle the balance of quality of life and the adequate treatment of the complications of dementia. Indeed, we see this in the drive to deprescribe medications that are potentially inappropriate medications (PIMs) that may not be appropriate for an older population.

Given that most geriatricians are not located in rural areas [12], clinical video telehealth (CVT) can be used to remotely diagnose and manage these patients. It has already been used to diagnose dementia [13], and has been implemented into practice for rural populations with dementia [14]. For instance, there exist programs in the U.S. with Veterans Affairs (VA) physicians being assisted with medication recommendations [15], as well as programs in other countries such as Canada in their rural and remote memory clinic (RRMC) which helped reduce the anticholinergic medication burden on dementia patients [16,17], and in Australia [18] where its use can overcome some barriers to provide adequate care, particularly in remote areas.

However, as many studies looking at rural populations with dementia using CVT often deal with the initial diagnosis, there is little data on the continued management of dementia patients in a rural setting using telemedicine, nor is there much data looking at what kinds of changes are made to affect those patients that have continued CVT management for dementia.

The VA Pittsburgh Healthcare System CVT program for dementia (Teledementia clinic) was started in 2013, serving veterans who are rural patients with cognitive decline that were unable to travel to the main campus either because the distance was too great or because they were too ill. Given that this population required dementia expertise that may not be readily accessible to those patients living outside a metropolitan area, CVT has been offered to help these rural veterans with dementia get care that optimized their medications to improve their quality of life. 

In addition to polypharmacy, which is estimated to occur in nearly 50% of older adults [19], there is the issue of underprescribing, or not having a medication to treat a medical condition without contraindication. Indeed, this is another frequent problem that is seen in the elderly [20] and can just as easily lead to poorer outcomes. To that end, we analyzed the patient visit data from our CVT consult program that was able to optimize the medications that are prescribed to these patients by looking at the medication changes recommended by our service between our new consults and our follow-up visits. We hypothesize that a medication list that more adequately balances the treatment of medical conditions and quality of life would be reflected by fewer changes per encounter as patients follow up with our service, which would provide more evidence of CVT’s usefulness in the care of dementia patients in a rural setting.

## 2. Materials and Methods

### 2.1. Teledementia Consult Service

The teledementia consult service consists of physicians trained in either geriatric medicine or psychiatry, a nurse practitioner to assist with medical assessments, clinical psychologists to assess cognitive function, and social workers to support the patient and their caretakers with referrals to services provided by the VA or community [21]. Patients are referred to our service via primary care physicians, often in Community-Based Outpatient Clinics (CBOCs), or via other specialists who believe that the patient can benefit from more integrated care of their cognitive impairment. These patients are usually within 30 min of a CBOC, though some can be farther away [22]. For remote consults in 2016, there were 8 CBOCs (Westmoreland, Washington, Fayette, Belmont, Beaver, Huntingdon, State College and Johnstown) and one rural VA hospital (Altoona) that referred patients to our service.

**Figure 1:**
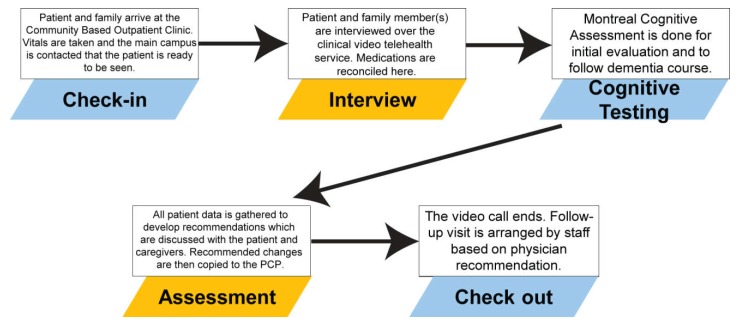
Structure of a Clinical Video Telehealth Encounter.

For each CVT appointment, which can either be a new patient consult or a follow-up visit, the structure for of these appointments are the same (Figure 1). These patients will present to a special clinic room in the CBOC where there is a monitor and camera system already set up. Once the patient is roomed and triaged, two-way communication via CVT is set up between the patient in the CBOC and the geriatric healthcare provider at the Veterans Affairs Pittsburgh Healthcare System main campus. A medical history is completed, followed by medication reconciliation, and if needed, cognitive screening which is most often a Montreal Cognitive Assessment [23]. Following the interview and screening, recommendations are discussed with the patient and their caregivers. In addition, these recommendations are forwarded in writing to their primary care physicians within the VA system via the VA’s electronic medical record known as the Computerized Patient Record System (CPRS).

### 2.2. Teledementia Database

Following each visit, data is entered into a secure Microsoft Access database containing demographic data of the patient (age, gender, reported ethnic group), recommendations of medications to be added, stopped or changed, as well as a brief summary of overall recommendations. This database combines changes made by both geriatric medicine and psychiatric portions of the consultation. Data is maintained by a single nurse, ensuring consistent data entry for each patient visit.

### 2.3. Study Characteristics

Our study is a retrospective observational study using the data abstracted from the teledementia database. We limited our initial data set to patient CVT sessions from 1 January–31 December 2016, separating the encounters into initial consults and follow-up visits. Medication recommendation data from the database was extracted for each of these encounters. The demographics are detailed in Table 1.

Our primary outcome was comparing the difference between initial consults and follow-up visits for medication additions (medications added that were not previously on the patient’s medication list), stops (medications discontinued that were previously on the patient’s medication list), dosage/timing modifications (medications that remained on the medications list before and after the visit, but whose dosage and/or timing was changed), overall medication changes (totaling all of the medication adds, stops and modifications in the encounter), and net medication change per encounter (medication adds subtracted by medication stops). In addition, we compared the difference between initial consults and follow-up visits for stops and dosage/timing modifications of PIMs found in the 2015 Beers Criteria [24]. We separated these medications into PIMs in all circumstances (Table 1 PIMs) and all PIMs found in the Beers Criteria including conditional PIMs (e.g., drug–drug interactions, renal function).

These outcomes were used as surrogates for medication optimization. We hypothesize that as a medication list becomes more effectively balanced between side effects with treatment of disease, the number of medication changes should be reduced in follow-up visits. Since older patients have issues with both polypharmacy and underprescribing, we felt that it was important to look at each different kind of medication change to better describe any changes that the CVT consults are having on prescribing patterns.

The per encounter data was coded by a single researcher in Microsoft Excel, with each encounter being anonymized of patient identifiers. Once coded, the averages of each of the above outcomes were calculated in both the initial consults and the follow-up visits. The averages were then analyzed via two-tailed t-test to detect statistical differences between the two groups using a null hypothesis of no difference. The data was manually reinputted and reanalyzed three months later to ensure that the initial analysis was correct.

Because this study was a quality improvement project in the Pittsburgh VA, it was exempt from Institutional Review Board review.

## 3. Results

### 3.1. Population Characteristics

The population represented in our sample of veterans in the CVT consult service were exclusively male. In addition, the majority were Caucasian in both initial consults (93.9%) and follow-up visits (92.8%). The racial and ethnic demographics of the patients were statistically similar; however, there was a higher percentage of patients that were seen by geriatric psychiatry in the follow-up visits than the initial consult (76.8% vs. 50%, *p* = 0.0002). Geriatric psychiatry involvement in these cases ranged from specialized management of significant psychiatric symptoms to titration of multiple psychotropic medications.

In our data set, we had 199 total encounters. Of these encounters, 130 were initial CVT consults and 69 were follow-up visits. In total, there were 308 total medication change events, with 230 events (74.7%) in the initial consults and 78 events (25.3%) in the follow-up visits.

In the 230 medication events of the initial consults, there were 95 medication adds (41.3%), 89 medication stops (38.7%), and 46 medication dosage/timing modifications (20%). In the 78 events of the follow-up visits, there were 30 medication adds (38.5%), 28 medication stops (35.9%), and 20 medication dosage/timing modifications (25.6%) (Table 2).

### 3.2. Medication Changes Overall

To measure our primary outcome measure, we found per encounter averages for each type of medication change (Table 3). For initial consults, we found an average of 0.731 medications added per encounter, 0.685 medications stopped per encounter, and 0.354 medications modified per encounter. The average overall medications changed per encounter was 1.769 in these initial consults. We also found that when all changes were taken into account, the initial encounters added an average of 0.046 medications to the patient’s medication list.

For the follow-up visits, we found fewer changes in every type. We found 0.435 medications added, 0.406 medications stopped, 0.290 medications modified, 1.130 total medications changed, and a net of 0.029 medications added per encounter.

When comparing the initial consults and the follow-up visits, we found that only the medications added (0.731 vs. 0.435, *p* = 0.0009) and the total medication changes (1.769 vs. 1.130, *p* = 0.0079) had reached statistical significance when compared between the initial consults and the follow-up visits. The medications stopped (0.686 vs. 0.0406, *p* = 0.0704), modifications (0.354 vs. 0.290, *p* = 0.4653) and net medication changes (0.046 vs. 0.029, *p* = 0.9158) did not reach statistical significance.

We had broken down the medications added, stopped and modified by types of medications to see if any particular type was driving the changes that we see in Table 2. Unfortunately, we were unable to find a single category of medication whose changes in either adding, stopping or modifications were statically significant between the initial consult and follow-up visit (Table A1).

### 3.3. Beers Criteria Medications

The next step of our analysis was to see if 2015 Beers Criteria medications were statistically different when the discontinuation or modifications were compared between the initial consult and the follow-up visits (Table 4). We found that Table 1 PIMs were statistically significant in both stopping (0.208 vs. 0.072, *p* = 0.025) and dosage/timing modification (0.230 vs. 0.029, *p* = 0.001). We also saw statistical significance when looking at all Beers List medications stopped (0.323 vs. 0.150, *p* = 0.044) though not when looking at dosage/timing modifications (0.338 vs. 0.202, *p* = 0.123). These results show that, for the most part, teledementia services for rural veterans had an effect on Beers List modifications, presumably with the lower numbers in the follow-up visits compared to the initial visit, indicating that the medication list was either optimized with the correct modifications of the Beers List medications and that those modifications, whether changes in dosage/timing or stopping all together, appeared to be carried through to the follow-up visit.

## 4. Discussion

Overall, the significance found in adds and total medications indicates that a greater number of changes happen in the initial visit and its effects on the medication list is evidence that the medications of the dementia patient were being optimized. Although it was disappointing to see that there was no statistical significance with stops, modifications, or net medication changes, it may be reflective of medications already being removed or changed prior to being seen in our CVT consult service. We do not see a statistically significant increase in any changes category, which can be seen as evidence that the CVT is not adversely affecting the patients’ medication regimens.

In addition, the decrease of stops and modification of PIMs between the initial consults and the follow-up visits is important as these PIMs can lead to increased delirium and other poor outcomes with older patients, particularly those medications that are psychogenic and whose side effects may be amplified by cognitive impairment.

One thing to note here is that there was no single drug class that showed significance either indicating that the sample size was not large enough or, more likely, that there were a large number of visits that resulted in 0 medication changes, greatly reducing the possibility of seeing an effect. At least in this population, while it does appear that there are, in aggregate, more medications being added in the initial consult versus the follow-up visit, there is no specific medication class that is noted to be driving this change.

Altogether, this data is encouraging in demonstrating that our teledementia service CVT consults can be used as a model to help with the management of medications in rural dementia patients. Previous research indicates that CVT for dementia patients can lead to a slowing of dementia progression [25,26]. It is possible that the effect that we see in previous studies may be related to the adequate treatment of dementia while deprescribing medications to increase quality of life and potentially reduce the risk of medication-induced delirium episodes. Indeed, the multidisciplinary team approach of caring for rural dementia patients using CVT includes social work [27] and physical therapy [28]. The data from our CVT consultation service providing psychiatric and dementia specialist support to rural primary care physicians is further evidence of effectively providing remote dementia care.

Given the shortages of geriatric expertise in rural areas, this can be a way to expand the reach of geriatric specialist care that may be more often found in urban areas. In the context of the VA, these kinds of CVT consults demonstrate further evidence of effectiveness in providing remote assistance to patients that may only be able to travel to a CBOC based on distance or other travel restrictions. This in turn may show our implementation to be a feasible model for treating rural dementia patients, as the expansion of telemedicine is a potential solution to practicing medicine in other low-resource environments [29,30,31].

Indeed, as the rural older adult population continues to grow, CVT may be another tool to help project the geriatric principles to these changing demographics, particularly in the face of a growing deficit of geriatricians [32] and other dementia specialists, particularly in rural areas. However, more research is required to define how best to effectively implement such a rural CVT program.

### 4.1. Limitations

There are several limitations to this study. First, as mentioned above, the large number of visits that had 0 medication changes resulted in average numbers that were less than 1. That combined with the low number of visits throughout the year analyzed made it difficult for some categories to approach significance.

Second, with a single team member doing analysis and coding, this may have led to some bias in how some of the data was interpreted. Although the definitions were clearly defined, it would have had more reliability if a second coder was employed to ensure that the interpretation of the data was reliable between multiple coders.

Third, our study design does not account for difference between other providers that are caring for the patient. For instance, a significantly higher percentage of patients in the follow-up visits were patients that were followed by geriatric psychiatry, as compared to the initial consults. This can confound the results as it is another team member on the consult service that can propose medication changes to the patients. We also do not take into account the training of the primary care clinicians (who may be physicians, nurse practitioners, or physician assistants) that follow the patient at the CBOC. In particular, we do not take into account clinicians who may be more comfortable changing medications between visits, further dampening the effect that our CVT consult has. Nevertheless, a future study should account for these confounding factors by comparing our CVT consults against a control group.

Lastly, although some of the patients were referred without having previously been seen by a geriatrician, some patients were referred to the teledementia consult service following a visit to the Geriatric Evaluation and Management (GEM) clinic in Pittsburgh. This interdisciplinary comprehensive geriatric assessment clinic consists of a visit from both a geriatrician as well as a pharmacist trained in geriatric principles, which may have dampened the medication change effect of the initial visit as the changes that would have normally been made during the CVT visit would have already been made.

### 4.2. Future Directions

Moving forward, we will include multiple years of data to increase the sample size. In addition, we would like to do a pre–post analysis by following the medication changes made for each patient between their initial consult and follow-up visits, so that the changes are made in context to each individual patient. By linking that data to each patient, we will also be able to better abstract from the electronic medical record hospitalizations, nursing home transfers, or medication adverse reactions as other outcome measures to compare.

## Figures and Tables

**Table 1:** Demographic Data of 2016 Teledementia Study Population.

Characteristics	Initial Consult	Follow-Up Visit	*p* Value
Gender, n (%)			N/A
Male	130 (100)	69 (100)	
Race, n (%)			0.952
Caucasian	122 (93.9)	64 (92.8)	
Black	5 (3.8)	3 (4.3)	
Declined to state	3 (2.3)	2 (2.9)	
Ethnicity, n (%)			0.089
Hispanic	1 (0.8)	0 (0)	
Not hispanic	127 (97.7)	65 (92.8)	
Declined to state	2 (1.5)	5 (7.2)	
Seen by geriatric psychiatry, n (%)			0.0002 **
Yes	65 (50)	53 (76.8)	
No	65 (50)	16 (23.2)	

** *p* < 0.01.

**Table 2:** CVT encounters and medication events by type.

Characteristics	Initial Consult	Follow-Up Visit	Combined
CVT Encounters	130	69	199
Overall medication events	230	78	308
Total medication add events, n (%)	95 (41.3)	30 (38.5)	125 (40.6)
Total medication stop events, n (%)	89 (38.7)	28 (35.9)	117 (38.0)
Total medication modification events, n (%)	46 (20.0)	20 (25.6)	66 (21.4)

**Table 3:** Overall medication additions, stops, modifications, and total changes per encounter.

Type of Medication Change Per Encounter	Initial Consult (N = 130)	Follow-Up Visit (N = 69)	*p* Value
Medications added	0.731	0.435	0.0009 **
Medications stopped	0.685	0.406	0.0704
Medications modified	0.354	0.290	0.4653
Total medication changes	1.769	1.130	0.0079 **
Net medications changed	0.046	0.029	0.9158

** *p* < 0.01.

**Table 4:** 2015 Beers Criteria medications stopped or modified per encounter.

Type of Medication Change	Initial Consult (N = 130)	Follow-Up Visit (N = 69)	*p* Value
2015 Beers Criteria Table 1 PIMs			
Medications stopped (per encounter)	0.208	0.001	0.025 *
Medications modified (per encounter)	0.230	0.029	0.001 **
All 2015 Beers Criteria medications			
Medications stopped (per encounter)	0.323	0.150	0.044 *
Medications modified (per encounter)	0.338	0.202	0.123

* *p* < 0.05, ** *p* < 0.01.

## Supplementary Materials

Supplementary File 1

## Appendix A

**Table A1:** Medication additions, stops and modifications by medication category.

Medication Changes by Type	Initial Consult	Follow-Up Visit	*p* Value
Type of medication added per encounter			
Anti-dementia	0.177	0.101	0.1583
Antidepressants	0.185	0.203	0.7703
Pain	0.054	0.000	0.0501
Antipsychotics	0.038	0.014	0.3492
Anticholinergics	0.008	0.000	0.4677
Supplements	0.200	0.101	0.0974
Type of medication stopped per encounter			
Anti-dementia	0.069	0.043	0.5057
Antidepressants	0.077	0.072	0.9209
Pain	0.031	0.000	0.1409
Antipsychotics	0.000	0.000	N/A
Anticholinergics	0.077	0.043	0.4023
Supplements	0.092	0.101	0.8813
Type of medication modified per encounter			
Anti-dementia	0.285	0.203	0.2943
Antidepressants	0.362	0.420	0.5531
Pain	0.092	0.014	0.0844
Antipsychotics	0.100	0.029	0.0715
Anticholinergics	0.085	0.043	0.3171
Supplements	0.300	0.203	0.2770

## References

[1] Mohr N.M., Young T., Harland K.K., Skow B., Wittrock A., Bell A., Ward M.M. (2018). Emergency Department Telemedicine Shortens Rural Time-to-Provider and Emergency Department Transfer Times. Telemed. J. E Health.

[2] Perry T.T., Halterman J.S., Brown R.H., Luo C., Randle S.M., Hunter C.R., Rettiganti M. (2018). Results of an asthma education program delivered via telemedicine in rural schools. Ann. Allergy Asthma Immunol..

[3] (2017). World Health Organization: Dementia Fact Sheets.

[4] Wimo A., Guerchet M., Ali G.C., Wu Y.T., Prina A.M., Winblad B., Jonsson L., Liu Z., Prince M. (2017). The worldwide costs of dementia 2015 and comparisons with 2010. Alzheimers Dement..

[5] Wimo A., Jonsson L., Bond J., Prince M., Winblad B., Alzheimer Disease International (2013). The worldwide economic impact of dementia 2010. Alzheimers Dement..

[6] Leelakanok N., D’Cunha R.R. (2018). Association between polypharmacy and dementia—A systematic review and meta-analysis. Aging Ment. Health.

[7] Kristensen R.U., Norgaard A., Jensen-Dahm C., Gasse C., Wimberley T., Waldemar G. (2018). Polypharmacy and Potentially Inappropriate Medication in People with Dementia: A Nationwide Study. J. Alzheimers Dis..

[8] Masumoto S., Sato M., Maeno T., Ichinohe Y. (2018). Potentially inappropriate medications with polypharmacy increase the risk of falls in older Japanese patients: 1-year prospective cohort study. Geriatr. Gerontol. Int..

[9] Price S.D., Holman C.D., Sanfilippo F.M., Emery J.D. (2014). Association between potentially inappropriate medications from the Beers criteria and the risk of unplanned hospitalization in elderly patients. Ann. Pharmacother..

[10] Mackenzie T.A., Wallace A.E., Weeks W.B. (2010). Impact of rural residence on survival of male veterans affairs patients after age 65. J. Rural Health.

[11] (2013). U.S. Census Comparative Demographic Estimates: 2013 American Community Survey 1-Year Estimates. https://factfinder.census.gov/faces/tableservices/jsf/pages/productview.xhtml?src=bkmk.

[12] Section for Enhancing Geriatric Understanding and Expertise Among Surgical and Medical Specialists (SEGUE), American Geriatrics Society (2011). Retooling for an Aging America: Building the Healthcare Workforce. A white paper regarding implementation of recommendation 4.2 of this Institute of Medicine Report of April 14, 2008, that “All licensure, certification and maintenance of certification for healthcare professionals should include demonstration of competence in care of older adults as a criterion.”. J. Am. Geriatr. Soc..

[13] Martin-Khan M., Flicker L., Wootton R., Loh P.K., Edwards H., Varghese P., Byrne G.J., Klein K., Gray L.C. (2012). The diagnostic accuracy of telegeriatrics for the diagnosis of dementia via video conferencing. J. Am. Med. Dir. Assoc..

[14] Dang S., Gomez-Orozco C.A., van Zuilen M.H., Levis S. (2018). Providing Dementia Consultations to Veterans Using Clinical Video Telehealth: Results from a Clinical Demonstration Project. Telemed. J. E Health.

[15] Vandenberg A.E., Echt K.V., Kemp L., McGwin G., Perkins M.M., Mirk A.K. (2018). Academic Detailing with Provider Audit and Feedback Improve Prescribing Quality for Older Veterans. J. Am. Geriatr. Soc..

[16] Steve T.A., Kirk A., Crossley M., Morgan D., D’Arcy C., Biem J., Forbes D., Stewart N. (2008). Medication use in patients presenting to a rural and remote memory clinic. Can. J. Neurol. Sci..

[17] Verity R., Kirk A., Morgan D., Karunanayake C. (2016). Trends in Medication Use Over 11 Years in Patients Presenting to a Rural and Remote Memory Clinic. Can. J. Neurol. Sci..

[18] Van Ast P., Larson A. (2007). Supporting rural carers through telehealth. Rural Remote Health.

[19] Maher R.L., Hanlon J., Hajjar E.R. (2014). Clinical consequences of polypharmacy in elderly. Expert Opin. Drug Saf..

[20] Kuijpers M.A., van Marum R.J., Egberts A.C., Jansen P.A. (2008). Relationship between polypharmacy and underprescribing. Br. J. Clin. Pharmacol..

[21] Powers B.B., Homer M.C., Morone N., Edmonds N., Rossi M.I. (2017). Creation of an Interprofessional Teledementia Clinic for Rural Veterans: Preliminary Data. J. Am. Geriatr. Soc..

[22] Hedeen A.N., Heagerty P.J., Fortney J.C., Borowsky S.J., Walder D.J., Chapko M.K. (2002). VA community-based outpatient clinics: Quality of care performance measures. Med. Care.

[23] Nasreddine Z.S., Phillips N.A., Bédirian V., Charbonneau S., Whitehead V., Collin I., Cummings J.L., Chertkow H. (2005). The Montreal Cognitive Assessment, MoCA: A brief screening tool for mild cognitive impairment. J. Am. Geriatr. Soc..

[24] American Geriatrics Society 2015 Beers Criteria Update Expert Panel (2015). American Geriatrics Society 2015 Updated Beers Criteria for Potentially Inappropriate Medication Use in Older Adults. J. Am. Geriatr. Soc..

[25] Cheong C.K., Lim K.H., Jang J.W., Jhoo J.H. (2015). The effect of telemedicine on the duration of treatment in dementia patients. J. Telemed. Telecare.

[26] Kim H., Jhoo J.H., Jang J.W. (2017). The effect of telemedicine on cognitive decline in patients with dementia. J. Telemed. Telecare.

[27] Bryant L., Garnham B., Tedmanson D., Diamandi S. (2018). Tele-social work and mental health in rural and remote communities in Australia. Int. Soc. Work.

[28] Burton R.L., O’Connell M.E. (2018). Telehealth Rehabilitation for Cognitive Impairment: Randomized Controlled Feasibility Trial. JMIR Res. Protoc..

[29] Durrani H., Khoja S. (2009). A systematic review of the use of telehealth in Asian countries. J. Telemed. Telecare.

[30] Sarfo F.S., Adamu S., Awuah D., Ovbiagele B. (2017). Tele-neurology in sub-Saharan Africa: A systematic review of the literature. J. Neurol. Sci..

[31] Lee J.H., Kim J.H., Jhoo J.H., Lee K.U., Kim K.W., Lee D.Y., Woo J.I. (2000). A telemedicine system as a care modality for dementia patients in Korea. Alzheimer Dis. Assoc. Disord..

[32] Institute of Medicine (US) Committee on the Future Health Care Workforce for Older Americans (2008). Retooling for an Aging America: Building the Health Care Workforce.

