# Supplementary figures and images for: Use of Clinical Video Telehealth as a Tool for Optimizing Medications for Rural Older Veterans with Dementia
Source: Geriatrics (Basel). 2018 Jul 30;3(3):44. doi: 10.3390/geriatrics3030044 (PMC6319231; doi:10.3390/geriatrics3030044)

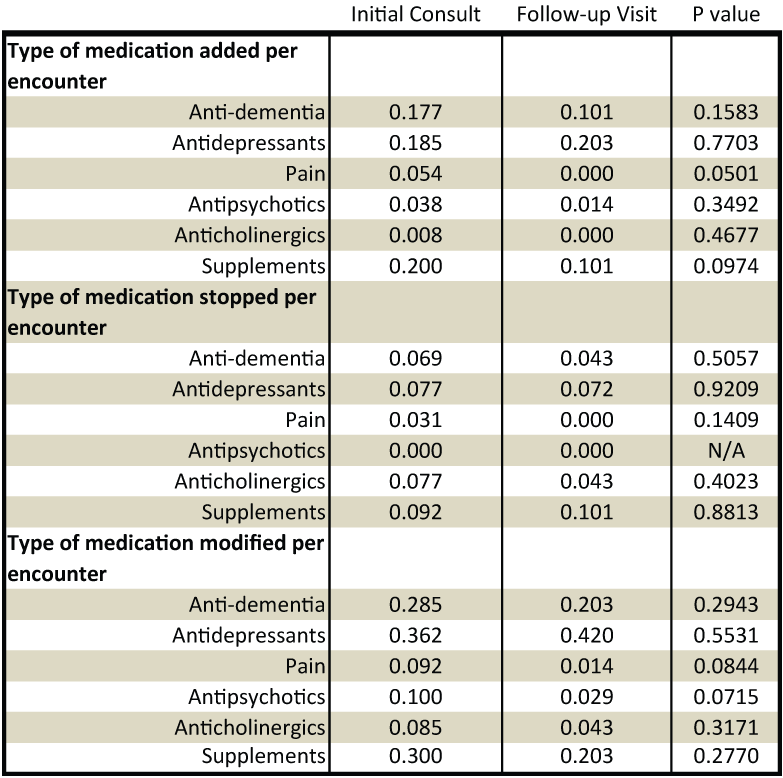

Supplement: Supplementary File 1 [file geriatrics-03-00044-s001.zip › Supplemental.tif]
